# OP39 Health Technology Assessment In Developing Countries: Challenges And Advances. A Survey Conducted By The Developing Countries Interest Group

**DOI:** 10.1017/S0266462325100974

**Published:** 2025-12-29

**Authors:** Anthony Kamau, Gladys Ukamaka, Magdalena Moshi, Kavita Kachroo, Marilla Cardoso, Marisa Santos, Yanga Nokhepheyi, Meera Muthukrishnan, Appiah Obeng, Debjani Mueller

## Abstract

**Introduction:**

Health technology assessment (HTA) has gained increased attention and investment in low- and middle-income countries (LMICs). HTAi Interest Groups provide critical platforms for networking, information exchange, and collaborative research. To understand the challenges and advances in HTA across LMICs, the Developing Countries Interest Group (DCIG) conducted a comprehensive survey in 2024.

**Methods:**

The survey targeted LMICs, focusing on the following dimensions of HTA: operational frameworks (geographical distribution, focus areas, patient engagement, rare disease processes) and a SWOT analysis assessing: (i) strengths—things that the country does particularly well or that distinguishes it from others; (ii) weaknesses—features, resources, systems, and procedures, including what could improve and the sort of practices should be avoided; (iii) opportunities—openings or chances for something positive to happen; and (iv) threats—any issue that can negatively affect the development. Additionally, respondents identified key challenges in implementing HTA and proposed ways the HTAi DCIG could address these barriers.

**Results:**

The survey received 33 responses, with 50 percent being HTAi members, 36.3 percent from Latin America, 27.2 percent from Africa, and others from Asia. Of the respondents, 57.6 percent reported having an HTA department in their ministry of health, 48.5 percent had a national policy, and 84.4 percent used HTA for assessing medicines and devices. However, 54.5 percent lacked patient engagement processes. Genuine interest in building local capacity for HTA, but weaknesses included knowledge gaps, with limited funding and awareness among stakeholders as significant threats. Opportunities arose from growing interest among health professionals. Respondents suggested the DCIG support HTA institutionalization and provide foundational training.

**Conclusions:**

The findings underscored active HTAi member engagement, especially in Latin America, Asia and Africa. While many countries have established HTA departments and policies, gaps persist in patient engagement and knowledge dissemination. Key strengths included interest in capacity building, while weaknesses included knowledge gaps. Opportunities for growth exist, but limited funding poses a threat. Respondents recommended that the DCIG provide support in institutionalizing HTA and training.

